# Extensive Reduction of the Nuclear Pore Complex in Nucleomorphs

**DOI:** 10.1093/gbe/evz029

**Published:** 2019-02-04

**Authors:** Nicholas A T Irwin, Patrick J Keeling

**Affiliations:** Department of Botany, University of British Columbia, Vancouver, British Columbia, Canada

**Keywords:** nuclear pore, nucleomorph, chlorarachniophytes, cryptophytes, endosymbiosis, endosymbiotic gene transfer

## Abstract

The nuclear pore complex (NPC) is a large macromolecular assembly situated within the pores of the nuclear envelope. Through interactions between its subcomplexes and import proteins, the NPC mediates the transport of molecules into and out of the nucleus and facilitates dynamic chromatin regulation and gene expression. Accordingly, the NPC constitutes a highly integrated nuclear component that is ubiquitous and conserved among eukaryotes. Potential exceptions to this are nucleomorphs: Highly reduced, relict nuclei that were derived from green and red algae following their endosymbiotic integration into two lineages, the chlorarachniophytes and the cryptophyceans. A previous investigation failed to identify NPC genes in nucleomorph genomes suggesting that these genes have either been relocated to the host nucleus or lost. Here, we sought to investigate the composition of the NPC in nucleomorphs by using genomic and transcriptomic data to identify and phylogenetically classify NPC proteins in nucleomorph-containing algae. Although we found NPC proteins in all examined lineages, most of those found in chlorarachniophytes and cryptophyceans were single copy, host-related proteins that lacked signal peptides. Two exceptions were Nup98 and Rae1, which had clear nucleomorph-derived homologs. However, these proteins alone are likely insufficient to structure a canonical NPC and previous reports revealed that Nup98 and Rae1 have other nuclear functions. Ultimately, these data indicate that nucleomorphs represent eukaryotic nuclei without a canonical NPC, raising fundamental questions about their structure and function.

## Introduction

The nuclear pore complex (NPC) is a large macromolecular assembly that structures the membranous pores of the nuclear envelope and serves as a gate into the nucleus (Strambio-De-Castillia et al. 2010; Beck and Hurt 2017). The NPC consists of over 30 protein subunits, termed nucleoporins, that are typically arranged with 8-fold symmetry around the central channel of the pore. These subunits are organized into individual subcomplexes: the cytoplasmic complex, which regulates nuclear import and export; the outer, inner, and transmembrane rings, which form structural scaffolds; the central channel, which mediates passage through the pore; and the nuclear basket, which interacts with nuclear factors (Beck and Hurt 2017). These subcomplexes act in concert to regulate nucleocytoplasmic exchange through two mechanisms (Stewart 2007). First, the NPC generates a size exclusion barrier that bars the passive diffusion of molecules >40 kDa (Paine et al. 1975). Second, the NPC mediates the passage of materials exceeding this threshold through an active transport system dependent on nuclear localization signals, transport receptors, and import proteins (Stewart 2007). Through these mechanisms, the NPC facilitates the bidirectional exchange of proteins into and out of the nucleus. But nucleoporins can also function beyond trafficking. Indeed, nucleoporins facilitate dynamic chromatin regulation and gene expression through trafficking-independent mechanisms by manipulating genome architecture and activating transcription (Capelson et al. 2010; Liang and Hetzer 2011; Bermejo et al. 2012). Therefore, the NPC not only represents a trafficking hub but also a highly integrated nuclear component that is ubiquitous and highly conserved among eukaryotes (Neumann et al. 2010).

One potential exception to the conservation of the NPC are nucleomorphs. Nucleomorphs are highly reduced, relict nuclei that were derived by secondary endosymbiosis (Greenwood 1974; Ludwig and Gibbs 1989; McFadden and Gilson 1995; Gilson et al. 2006; Archibald 2007). In contrast to primary endosymbiosis, where a bacterium is incorporated into a eukaryotic cell, secondary endosymbiosis involves the uptake of one eukaryote by another, or more specifically, the uptake of a primary alga such as a green or red alga by another eukaryote (Keeling 2013). Secondary endosymbiosis typically results in the complete reduction of the endosymbiont, leaving only the plastid; however, in two instances, the nucleus of the endosymbiont has remained (Keeling 2013). These nuclei, termed nucleomorphs, were retained in two lineages, the chlorarachniophytes and the cryptophyceans (plastid containing cryptista) (Greenwood 1974; Ludwig and Gibbs 1989). The nucleomorphs of chlorarachniophytes and cryptophyceans were derived independently from green and red alga, respectively. However, these structures exhibit strong convergent reductive evolution both in terms of genome organization and nuclear functionality. For example, both chlorarachniophyte and cryptophycean nucleomorph genomes house three small linear chromosomes and typically conserved nuclear features such as histone posttranslational modifications and the C-terminal domain of RNA polymerase II have been highly reduced or lost in both instances (Douglas et al. 2001; Gilson et al. 2006; Marinov and Lynch 2016). Consequently, nucleomorphs represent a unique, highly reduced nuclear system, but whether this reduction has affected the structure and function of other conserved features, including nuclear pores, remains unclear.

A previous investigation into whether NPC genes are encoded in the nucleomorph genome itself failed to identify any homologs in both chlorarachniophyte and cryptophycean nucleomorphs (Neumann et al. 2006). This suggests at least three nonexclusive possibilities: 1) that nucleomorph NPC genes have been transferred to the host nucleus; 2) that host NPC proteins are dual targeted to both the host nucleus and nucleomorph; and 3) that the nucleomorph has lost its NPC (Neumann et al. 2006). To discern between these possibilities, we used nuclear genomic and transcriptomic data from chlorarachniophytes and cryptophyceans to identify and phylogenetically classify all NPC proteins in nucleomorph-containing algal lineages, as well as in red and green algal representatives, and nonphotosynthetic lineages closely related to the host. If nucleomorph NPC genes were transferred to the host nucleus, we would expect to identify two sets of nucleoporins: one of which would be of algal origin, and the other of host origin. If a dual-targeting mechanism had been employed, we would observe the expression of multiple isoforms of host homologs, some of which would contain the signal and transit peptides required for nucleomorph targeting (Gould et al. 2006; Hirakawa et al. 2009, 2010). Finally, if the nucleomorph lacks an NPC, then only host-derived genes should be found, comparable to outgroup taxa. Overall, this search supports the latter explanation: chlorarachniophytes and cryptophyceans mostly encode single, host-related NPC genes lacking signal peptides. Two exceptions were Nup98 and Rae1 which had clear nucleomorph-derived homologs, but these two proteins are insufficient to structure a canonical NPC alone and are known to have other, non-NPC functions in the nucleus. Ultimately, these data suggest that nucleomorphs represent eukaryotic nuclei that function without a canonical NPC.

## Materials and Methods

### Data Acquisition and Completeness Analysis

Transcriptomic data were obtained for 8 chlorarachniophytes (*Bigelowiella longifila*, MMETSP1359; *Norisiella sphaerica*, MMETSP0113; *Chlorarachnion reptans*, MMETSP0109; *Amorphochlora amoebiformis*, MMETSP0042; *Gymnochlora* sp., MMETSP0110; *Partenskyella glossopodia*, MMETSP1318; *Lotharella globosa*, MMETSP0111; and *Lotharella* sp., MMETSP0040), 1 rhizarian outgroup (*Ammonia* sp., MMETSP1384), and 11 cryptistans (*Palpitomonas bilix*, MMETSP0780; *Goniomonas* sp., MMETSP0114; *Goniomonas pacifica*, MMETSP0108; *Cryptomonas curvata*, MMETSP1050; *Cryptomonas paramecium*, MMETSP0038; *Hemiselmis virescens*, MMETSP1356; *Hemiselmis rufescens*, MMETSP1357; *Hemiselmis andersenii*, MMETSP0043; *Rhodomonas abbreviata*, MMETSP1101; *Rhodomonas lens*, MMETSP0484; and *Rhodomonas salina*, MMETSP1047) from the reassembled set of transcriptomes generated during the Marine Microbial Eukaryotic Transcriptome Sequencing Project (Keeling et al. 2014; Johnson et al. 2018). The previously generated transcriptome of *Paulinella chromatophora* (SRR3221671) was also utilized (Nowack et al. 2016). The genomes of *Bigelowiella natans*, *Plasmodiophora brassicae*, *Guillardia theta*, Cryptophyceae sp. CCMP2293, *Chlamydomonas reinhardtii*, *Volvox carteri*, and *Ostreococcus tauri* were downloaded from the Joint Genome Institute database (Merchant et al. 2007; Palenik et al. 2007; Prochnik et al. 2010; Curtis et al. 2012; Schwelm et al. 2015). The red algal genomes of *Cyanidioschyzon merolae* and *Chondrus crispus*, downloaded from EnsemblPlants, were also utilized (Nozaki et al. 2007; Collen et al. 2013). Proteins were predicted from the transcriptomic data using TransDecoder v5.1.0 (Haas et al. 2013). In order to assess the completeness of each data set, the presence of universal single copy orthologs was determined using Benchmarking Universal Single Copy Orthologs (BUSCO) v3.0.2 and the eukaryotic BUSCO database (Simão et al. 2015).

### Nucleoporin Identification

Although NPCs are conserved across the tree of eukaryotes, sequence divergence makes BLAST-dependent identification of nuclear pore proteins problematic (Neumann et al. 2010). To circumvent this, we used profile hidden Markov models (HMMs) to identify nuclear pore proteins based on domain structure as well as sequence composition, as has been successfully done in previous analyses (Neumann et al. 2010). Previously curated sets of nucleoporins from diverse eukaryotic taxa were obtained (Neumann et al. 2010) and realigned using the high accuracy L-INS-i algorithm of MAFFT v7.222 (Katoh and Standley 2013). Profile HMMs were generated using these alignments and HMM searches were conducted on all transcriptomes and genomes using HMMER v3.1 and an *E*-value threshold of 10^−5^ (Finn et al. 2011). The best 200 hits, which included all the hits (with the exception of Aladin, Nup37, Nup43, Rae1, Sec13, Seh1, and Tpr), were then extracted and incorporated into the original alignments and realigned as before. The resultant alignments were then used to generate phylogenies in IQ-Tree v.1.5.4 using the LG+G4 substitution model and statistical support was assessed using 1,000 ultrafast bootstrap pseudoreplicates (Nguyen et al. 2015; Hoang et al. 2018). Concurrently, all the proteins identified in the HMM searches were used as queries in position specific-iterative BLAST (PSI-BLAST) searches against the SWISS-PROT database (Altschul et al. 1997; Boeckmann et al. 2003). The phylogenies were then visualized in FigTree v1.4.2 and the operational taxonomic units were annotated with their best blast hit (*E*-value <10^−5^) to facilitate the removal of paralogous clades and the identification of true orthologs (Rambaut 2012). The trees were cleaned over three iterative cycles that involved removing obvious paralogs, realigning the sequences, and remaking the trees. Hits were considered true positives if they clustered with proteins from related species and/or if their best blast hit was the protein of interest. However, they were discarded if they clearly grouped with paralogs. Once the nucleoporins had been identified, long branching reference taxa were removed (typically parasitic species), and the remaining sequences were realigned and used to generate maximum likelihood phylogenies in IQ-Tree v.1.5.4 (Nguyen et al. 2015). Phylogenetic models were selected for each tree based on Bayesian Information Criteria using ModelFinder as implemented in IQ-Tree, and statistical support was assessed using 1,000 ultrafast bootstrap pseudoreplicates (Kalyaanamoorthy et al. 2017; Hoang et al. 2018). The finalized phylogenetic trees were visually inspected in FigTree v1.4.2. To ensure the reproducibility of the search and to check that nucleoporins had not been missed, the search was conducted twice using modified thresholds and identification criteria. In both cases, the same data sets were recovered. The phylogenetic affinity of each of the nucleoporins was then assessed using the finalized trees. Nucleoporins were considered host- or algal-related if they branched with outgroups or algae with an ultrafast bootstrap support over 70.

Once the NPC proteins had been identified, each was individually assessed for the presence of a signal peptide, a motif required for nucleomorph targeting (Hirakawa et al. 2009). Signal peptide predictions were performed using SignalP v.4.1 using the sensitive settings as well as SignalP v.3.0 using both the HMM and neural network–based approaches (Bendtsen et al. 2004; Petersen et al. 2011). In all cases, the same conclusions were drawn. Signal peptides are typically found at the N-termini of proteins and therefore, in order to facilitate the identification of false-negatives due to incomplete protein predictions, we also sought to determine whether or not each identified protein had a complete N-terminus. To this end, we used completeness predictions from TransDecoder and also manually inspected alignments to assess the presence of aligned N-terminal methionines (Haas et al. 2013). Besides false-negatives, SignalP also occasionally predicted signal peptides in proteins with incomplete N-terminal ends, suggesting that some predictions were false positives.

All the data obtained, including HMM profiles, identified nucleoporins, signal peptide prediction scores, alignments, phylogenies, and their associated models are available from Dryad (https://doi.org/10.5061/dryad.b0hs8gr) or from the authors, upon request.

### Pom152 Analysis

In order to more closely investigate the evolutionary history of Pom152, the alignment and tree were supplemented with prokaryotic homologs. Bacterial and archaeal reference proteomes were downloaded from UniProt (downloaded November 2016) and searched using the Pom152 HMM with an *E*-value cutoff of 10^−5^. The resulting hits were added to the Pom152 alignment and realigned using MAFFT L-INS-i (Katoh and Standley 2013). The phylogeny was generated as above using IQ-Tree and support was inferred from 1,000 ultrafast bootstrap pseudoreplicates.

## Results and Discussion

### Identification of Nucleoporins in Nucleomorph-Containing Algae and Their Relatives

To characterize and identify the origin of nucleomorph nucleoporins, we used profile HMMs, generated from alignments of previously identified NPC proteins from diverse eukaryotes (Neumann et al. 2010), to search for NPC proteins in the transcriptomes and genomes of nucleomorph-containing chlorarachniophytes and cryptophyceans (eight chlorarachniophyte transcriptomes, eight cryptophycean transcriptomes, one chlorarachniophyte genome, and two cryptophycean genomes; see Materials and Methods for details). We also examined three genomes from green algae and two from red algae, representing close relatives of the endosymbiotic partners of the chlorarachniophytes and cryptophyceans, respectively. Additionally, we examined two transcriptomes and a genome from nucleomorph-lacking relatives of chlorarachniophytes and three transcriptomes from nucleomorph-lacking cryptistans.

**Fig. 1.**
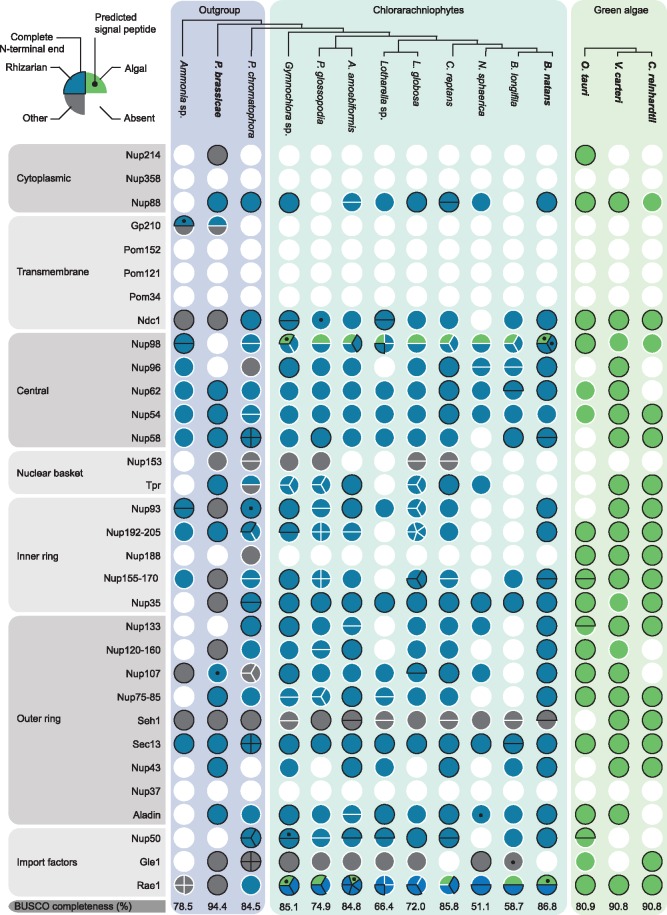
—Nucleoporins identified in nucleomorph-containing chlorarachniophytes, host-related lineages, and green algae. Circles represent proteins with each row corresponding to a different nucleoporin and each column representing a taxon. The different proteins are shown on the left and grouped into subcomplexes (Strambio-De-Castillia et al. 2010; Beck and Hurt 2017). Note that some proteins, such as Nup98, can be found in different parts of the NPC. A schematic phylogenetic tree is shown at the top and based on phylogenomic analyses (Irwin et al. 2019). Colored circles represent found proteins, whereas white circles represent absent proteins. Circles were subdivided to represent the number of protein copies that were identified and were colored based on the phylogenetic affinity of the protein. In particular, circles were colored blue, green, or gray depending on whether they clustered with rhizarian outgroups, algae or plants, or other taxa. Circles or wedges were outlined in black if they were predicted to have complete N-termini and black spots are present when a signal peptide was predicted using SignalP v4.1. Taxa with genome data have their names written in bold. The BUSCO completeness for each data set is provided in the last row.

**Fig. 2.**
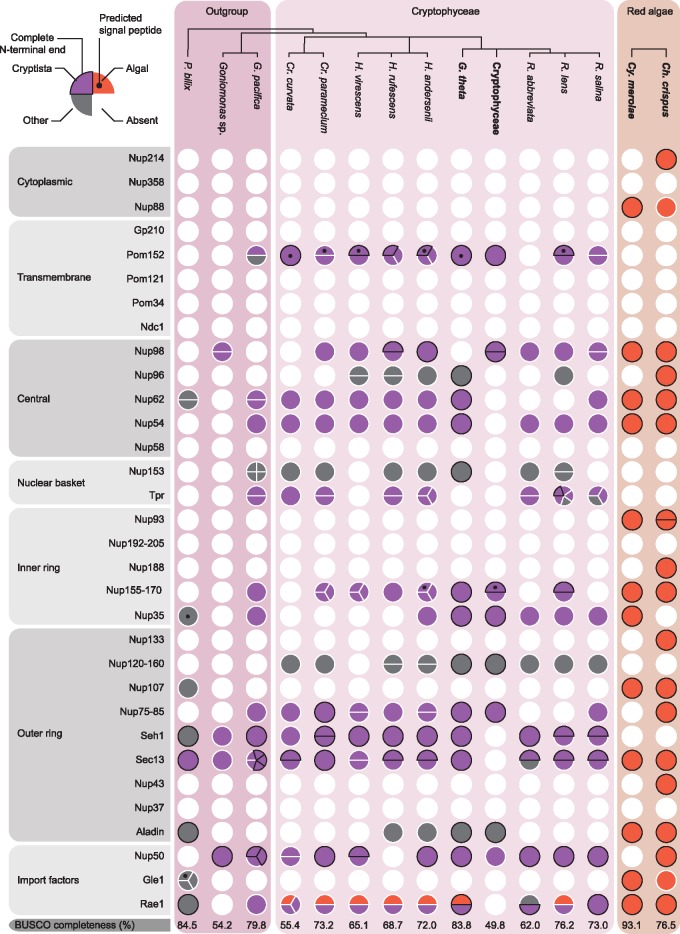
—Nucleoporins from cryptistans and red algae. For a complete description of this figure, see the figure legend for figure 1. A schematic phylogenetic tree is shown at the top and based on phylogenomic analyses (Burki et al. 2016). Circles were colored based on the phylogenetic affinity of the protein. In particular, circles were colored purple, red, or gray depending on whether they clustered with the cryptistan outgroups, algae or plants, or other taxa.

Our HMM search succeeded in identifying NPC proteins in all the examined lineages. Of the 32 nucleoporins, 27 were identified in chlorarachniophytes and their relatives (fig. 1 : 27 in outgroups and 24 in chlorarachniophytes) and 18 were found in cryptistans (fig. 2: 16 in outgroups and 16 in cryptophyceans). Furthermore, 25 NPC proteins were found in green algae and 19 in red algae. Animal and fungal-specific proteins, Nup37, Nup358, Pom34, and Pom121, were not identified in our data sets, highlighting the specificity of the search (Field et al. 2014). However, Pom152, traditionally considered to be a fungal-specific protein, was observed in 10 of the 13 cryptistan species. This protein was previously reported from the cryptophycean genome of *Guillardia theta* and was speculated to have been spread through horizontal gene transfer (Field et al. 2014). We sought to investigate this further by generating a phylogeny with cryptistan and fungal Pom152 proteins as well as prokaryotic homologs identified using the Pom152 HMM profile (supplementary fig. S1, Supplementary Material online). However, the resulting phylogeny was largely inconclusive, revealing a three-clade tree lacking obvious directionality.

Mapping the identified proteins to known NPC subcomplexes showed that the search results were comprehensive for both lineages. Within rhizarians (i.e., chlorarachniophytes and their relatives) and green algae, the identified nucleoporins corresponded to each of the NPC subcomplexes, whereas proteins structuring the cytoplasmic complex in cryptistans, and the transmembrane rings and nuclear basket in red algae, were conspicuously absent. BUSCO analysis revealed that transcriptomic data set completeness was variable but generally comparable to the genomic data sets, indicating that the data coverage was representative (figs. 1 and 2) (Simão et al. 2015). Furthermore, consistent absences in multiple taxa, independent of data set completeness, support the absence of these proteins (figs. 1 and 2). However, failure to identify homologs of subcomplex components does not necessarily mean the substructure itself is absent. Indeed, previous investigations into the structure of trypanosome NPCs identified novel nucleoporins and divergence in peripheral structures (i.e., the cytoplasmic complex and nuclear basket) (Holden et al. 2014; Obado et al. 2016). This was suggested to be a result of cytoplasmic and nuclear functional divergence which also could have occurred in some of the groups examined here (Obado et al. 2016).

Overall, the strongest trend in the data was the consistency of both copy number and phylogenetic affinity of the nucleoporins, suggesting that single isoforms of host origin predominate in both chlorarachniophytes and cryptophyceans (figs. 1 and 2). Multiple copies of some genes were identified in a few species, but they were exceptions and their narrow distribution suggests they are recent gene duplications, alternative transcripts, or misassemblies. Moreover, phylogenetic analysis showed that nearly all isoforms were derived from the host lineage and were not related to the algal endosymbiont (figs. 1 and 2). Consistent with this, almost no host-related proteins were predicted to contain signal peptides, with the exception of Pom152, a transmembrane nucleoporin. These data are therefore inconsistent with both the dual targeting of host proteins, and the large-scale transfer of NPC genes from the nucleomorph to the host genome.

The few nucleoporins that broke from this pattern were interesting exceptions, but none that suggested a nucleomorph NPC. First, several proteins were found to have an unresolved phylogeny. Nup96 was identified in five cryptophycean lineages as well as a red alga but was absent from cryptistan outgroups. Still, these cryptophycean sequences did not phylogenetically cluster with the endosymbiont lineage (fig. 2). Moreover, Nup153, Seh1, and Gle1 in the chlorarachniophytes, along with Nup153, Nup120-160, and Aladin in the cryptophyceans had uncertain origins due to phylogenetic ambiguities, or a lack of sampling of outgroups and/or algal proteins (figs. 1 and 2). Previous phylogenetic analyses have revealed that host-encoded nucleomorph genes do not necessarily cluster phylogenetically with the expected algal homologs, potentially because of artifacts such as long branch attraction (Gile and Keeling 2008; Hirakawa et al. 2011; Onuma et al. 2017). Hence, we cannot rule out that these nucleoporins are of nucleomorph origin. However, none of these proteins were predicted to contain signal peptides, which is expected for any protein targeted to the nucleomorph (Hirakawa et al. 2011), and some of those proteins, such as Seh1 and Gle1 in chlorarachniophytes, even branched with the outgroup lineages but with poor support (<70 ultrafast bootstrap), making the case for their nucleomorph localization and origin weak. Lastly, multiple isoforms of some nucleoporins (Nup192-205, Seh1, and Nup50 in chlorarachniophytes and Tpr and Sec13 in cryptophyceans) were observed in the majority of taxa. Although these proteins were not predicted to have signal peptides, many lacked complete N-terminal ends making the targeting of these proteins inconclusive. Despite this, the general lack of multiple isoforms and the absence of signal peptides strongly indicates that the widespread presence of host-derived nucleoporins in the nucleomorph is unlikely. Similarly, there is no evidence for the dual targeting of host nucleoporins based on alternative spicing or alternative transcription start sites. These genes would be very similar throughout the mature protein, so in principal could be easily overlooked. However, the improbability of overlooking the presence of alternative transcripts for every protein from every taxon analyzed makes this a weak explanation as well.

### Nup98 and Rae1 Are the Last Remaining Nucleomorph-Related NPC Proteins

**Fig. 3.**
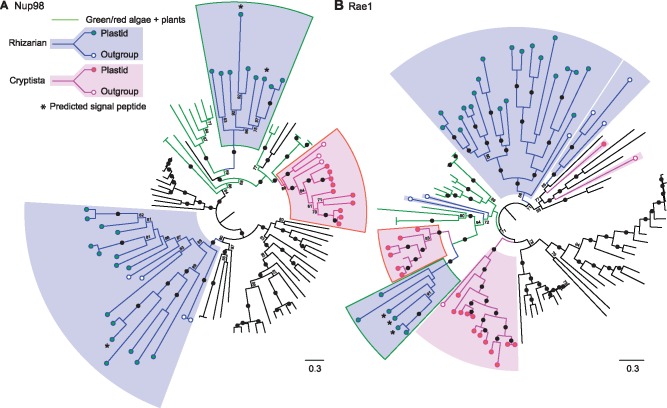
—Phylogenetic analysis of Nup98 and Rae1. Maximum likelihood phylogenies for Nup98 (*A*) and Rae1 (*B*) were generated in IQ-Tree using the VT+F+R5 model (Nup98) and the LG+F+R5 model (Rae1) as selected by ModelFinder. Statistical support was assessed using 1,000 ultrafast bootstraps and the resulting values are shown at the nodes. Values below 70 are not shown and black circles represent values over 95. Rhizarians, cryptistans, and algae and plants are labeled with blue, pink, and green, respectively. Rhizarian and cryptistan taxa containing nucleomorphs are denoted with green and red filled circles, whereas nucleomorph-lacking taxa are labeled with empty circles. Other eukaryotic taxa are shown in black and the trees were rooted at the midpoint. Host and endosymbiont-derived clades are outlined in white and green or red, respectively. The fully annotated trees are available in supplementary figure S2, Supplementary Material online.

In contrast to the ambiguous cases described above, two NPC proteins were clearly nucleomorph-related, Nup98 in chlorarachniophytes, and Rae1 in both chlorarachniophytes and cryptophyceans. Chlorarachniophytes consistently encoded multiple copies of both Nup98 and Rae1 (figs. 1 and 2), with one copy branching with the algal endosymbiont lineage and the other with outgroup taxa (fig. 3). Moreover, signal peptides were predicted in the algal-related Nup98 isoforms that had complete N-termini, and in three of the five algal-related Rae1 proteins (figs. 1 and 3). In cryptophyceans, an algal-related homolog of Rae1 was also found (fig. 2). Cryptophycean Rae1 genes were not predicted to encode signal peptides (fig. 2), which may reflect a relict nucleomorph protein that no longer undergoes trafficking but is more likely a misprediction because most of the algal-related homologs are also incomplete.

That both Nup98 and Rae1 would stand out is notable because both are also uniquely functionally diverse nucleoporins. Unlike most NPC proteins, Nup98 associates with either face of the pore and functions both at the nuclear envelope and within the nucleoplasm (Griffis et al. 2002, 2003). Similarly, Rae1 associates with the NPC but is also transient, facilitating mRNA export through interactions with microtubules and importins (Murphy et al. 1996; Blower et al. 2005). Nup98 is also known to play additional roles in diverse nuclear systems. For example, distinct Nup98 isoforms are used to distinguish the macronuclei and micronuclei of dikaryotic ciliates (Iwamoto et al. 2009), and in metazoans, Nup98 is important for NPC disassembly during mitosis (Laurell et al. 2011). Nup98 and Rae1 are also both known to function in RNA trafficking through mechanisms that are, at least in part, dependent on physical interactions between one another (Blevins et al. 2003; Blower et al. 2005; Ren et al. 2010). Furthermore, Nup98 and Rae1 form a cell cycle regulating complex capable of activating the anaphase promoting complex (APC), which facilitates cell cycle progression through the ubiquitin-dependent degradation of securins (Babu et al. 2003; Jeganathan et al. 2005). Given the functional diversity of these proteins, and in particular their functions outside the NPC, their role in the nucleomorph cannot be concluded to be NPC specific. However, Nup98 also associates with the central channel and the nucleomorph-related homologs still retain FG-repeat (Phenylalanine-Glycine repeat) domains, which are required for NPC function (Beck and Hurt 2017). Therefore, it is possible that Nup98 could be structuring a highly simplified NPC in chlorarachniophyte nucleomorphs.

A number of host-encoded nucleomorph-targeted proteins are implicated in cell cycle progression and have been predicted to provide the host with control over the division of its endosymbiont. These proteins include a DNA polymerase in chlorarachniophytes, as well as histones in both chlorarachniophytes and cryptophyceans (Hirakawa et al. 2011; Suzuki et al. 2016; Onuma et al. 2017). This is also possible for Nup98 and Rae1, however, neither nucleomorph-related Nup98 nor Rae1 is differentially expressed over the cell cycle in the chlorarachniophyte, *B.natans* (Suzuki et al. 2016), suggesting that if they are involved in the cell cycle, downstream regulators must be present. Furthermore, anaphase promoting complex proteins have not been identified in nucleomorph genomes and those annotated in the nuclear genome of *B. natans* are not predicted to be nucleomorph targeted (Curtis et al. 2012; Suzuki et al. 2016). Hence, the role of Nup98 and Rae1 in the chlorarachniophyte nucleomorph remains uncertain. The nucleomorph-related Rae1 in cryptophyceans is even more functionally ambiguous.

### A Nucleus without an NPC

**Fig. 4.**
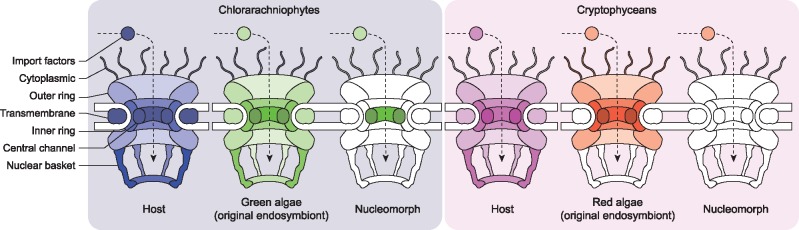
—NPC in chlorarachniophytes, cryptistans, their nucleomorphs, and their algal endosymbionts. A summary schematic depicting the different subcomplexes of the NPC. If a single protein was found corresponding to a given subcomplex, the structure was colored in. If no proteins were found, the subcomplex was left white.

The retention of only one or two clearly nucleomorph-derived NPC genes in both the chlorarachniophytes and cryptophyceans, and the lack of any evidence for the targeting of host-derived nucleoporins, reveals that nucleomorphs have convergently lost the molecular machinery required to structure a canonical NPC (fig. 4). Nucleomorphs are accordingly the only known eukaryotic nuclei that appear to function without a typical NPC, which is otherwise highly conserved (Neumann et al. 2010). However, the nucleus must maintain contact with the cytoplasm, and transmission electron microscopy has revealed porelike structures in the nucleomorph envelope (Ludwig and Gibbs 1989). Perhaps one clue to resolving this is in the observation that fluorescent fusion proteins targeted to the periplastidal compartment (or the endosymbiont-derived cytoplasm) of the chlorarachniophyte, *Amorphochlora amoebiformis*, localize to both the periplastidal compartment and the nucleomorph, despite exceeding the typical NPC size exclusion threshold of 40 kDa (Paine et al. 1975; Hirakawa et al. 2009). This suggests that there is a pore, but a functionally different one, possibly only facilitating passive transport and lacking the canonical regulatory capacity of the NPC. This would indicate that the nucleoplasm and cytoplasm were much more similar in these reduced systems than in their more complex ancestors.

In the absence of a canonical NPC (i.e., the specific and conserved set of proteins associated with pore), how a nucleomorph nuclear pore could be structured, particularly in cryptophyceans which lack Nup98, is unclear. Previous studies have revealed homology between nucleoporins and endomembrane components such as coatomers, which are a family of membrane-bending proteins, including those found on COPI and COPII-coated vesicles (Devos et al. 2004). It is thought that the NPC evolved by making these generic functions progressively more elaborate (Devos et al. 2004; Wilson and Dawson 2011). One hypothesis is that nucleomorph pores could have reverted to something akin to such an ancestral state, perhaps utilizing coatomer proteins for a much simpler structure involved only in membrane bending. It is also possible that some nucleomorph nucleoporins have been retained but in a highly derived state that cannot be detected with current bioinformatic methods. Moreover, nucleomorphs may utilize novel nucleoporins as is the case with trypanosomes, although even trypanosomes still retain the majority of the canonical NPC proteins (Neumann et al. 2010; Holden et al. 2014; Obado et al. 2016). Ultimately, biochemical analyses will be required to fully understand how nucleomorph pores are structured and function in such a reduced state.

Although the loss of the NPC is unique, the reduction in the complexity of protein trafficking systems may be a common occurrence during the reductive evolution of organelles. Reduced mitochondria, including hydrogenosomes and mitosomes, often lack seemingly essential mitochondrial import machinery such as the translocase of the inner membrane and translocase of the outer membrane complexes (Dolezal et al. 2005; Burri and Keeling 2007; Pyrihová et al. 2018). It is possible that trafficking proteins involved in nuclear transport could be similarly dispensable for baseline function, and thus the simplification of trafficking systems may represent a common theme in reductive evolution.

## Conclusions

Here, we show the nucleomorphs of chlorarachniophytes and cryptophyceans lack a canonical NPC as only two nucleomorph-derived nucleoporins, Nup98 and Rae1, could be identified in these lineages. These proteins are also involved in other cellular processes and are alone insufficient to structure a canonical NPC, revealing nucleomorphs to be the only known eukaryotic nuclei to lack the complex. Despite the likely retention of some kind of pore in the nucleomorph envelope, its exact structure, function, and role (if any) in regulation remain unknown. The lack of a canonical NPC suggests that investigations into the physical structure of nucleomorph pores should reveal unique insights into the function and evolution of the NPC as well as the reduction of endosymbionts and organelles.

## Supplementary Material

Supplementary data are available at *Genome Biology and Evolution* online.

## Supplementary Material

Supplementary Data
