# Supplementary figures and images for: Extensive Reduction of the Nuclear Pore Complex in Nucleomorphs
Source: Genome Biol Evol. 2019 Feb 4;11(3):678–87. doi: 10.1093/gbe/evz029 (PMC6411479; doi:10.1093/gbe/evz029)

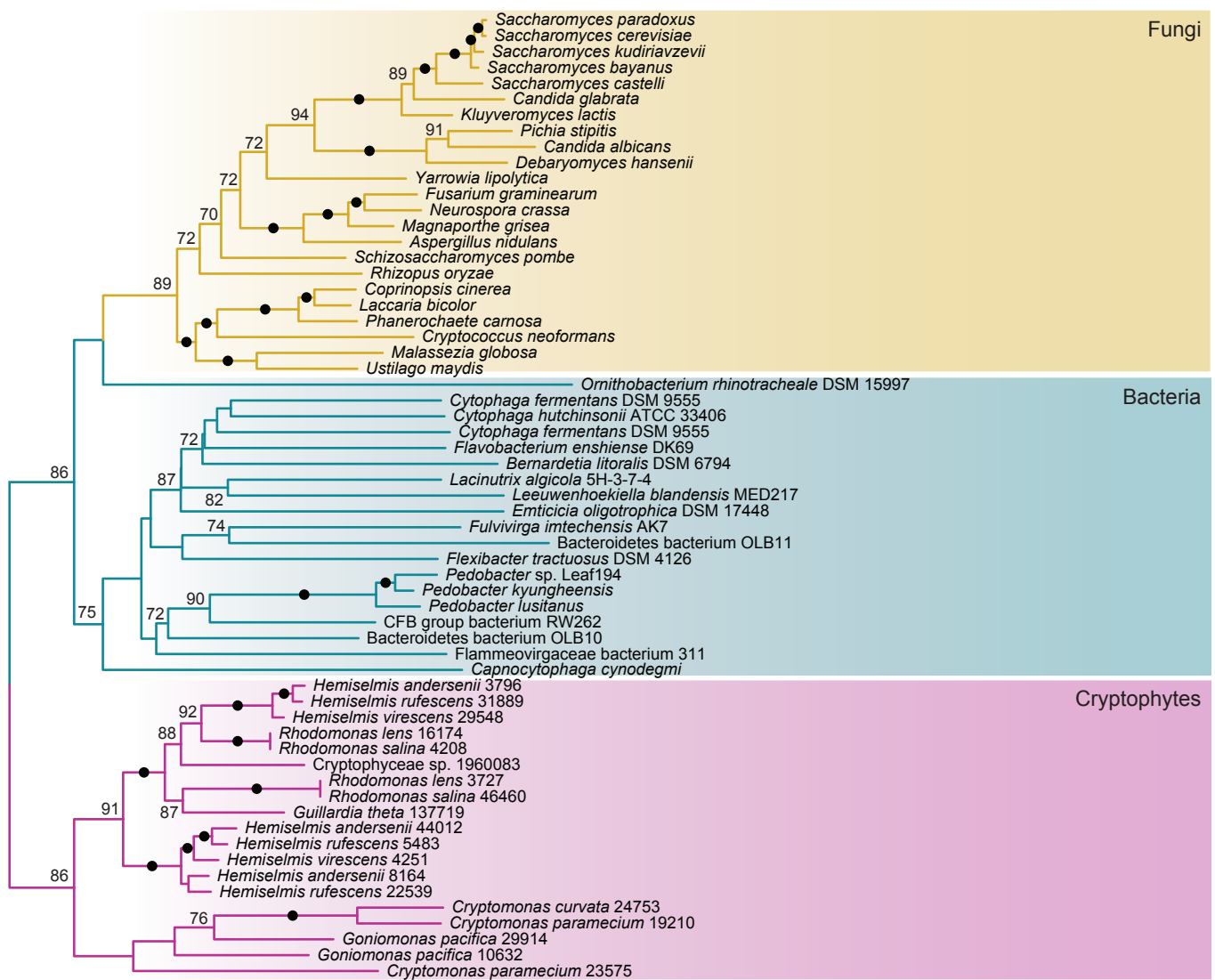

Supplement: Supplementary Data [file evz029_supp.zip › FigureS1.pdf]

A Nup98

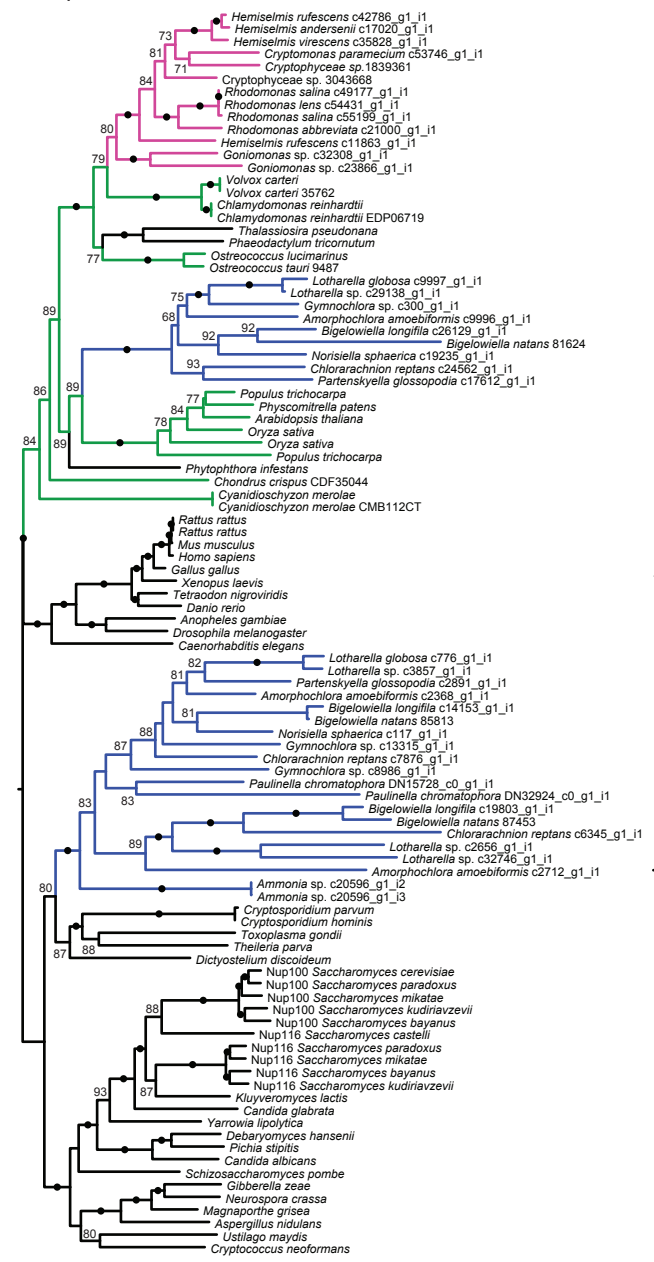

B Rae1

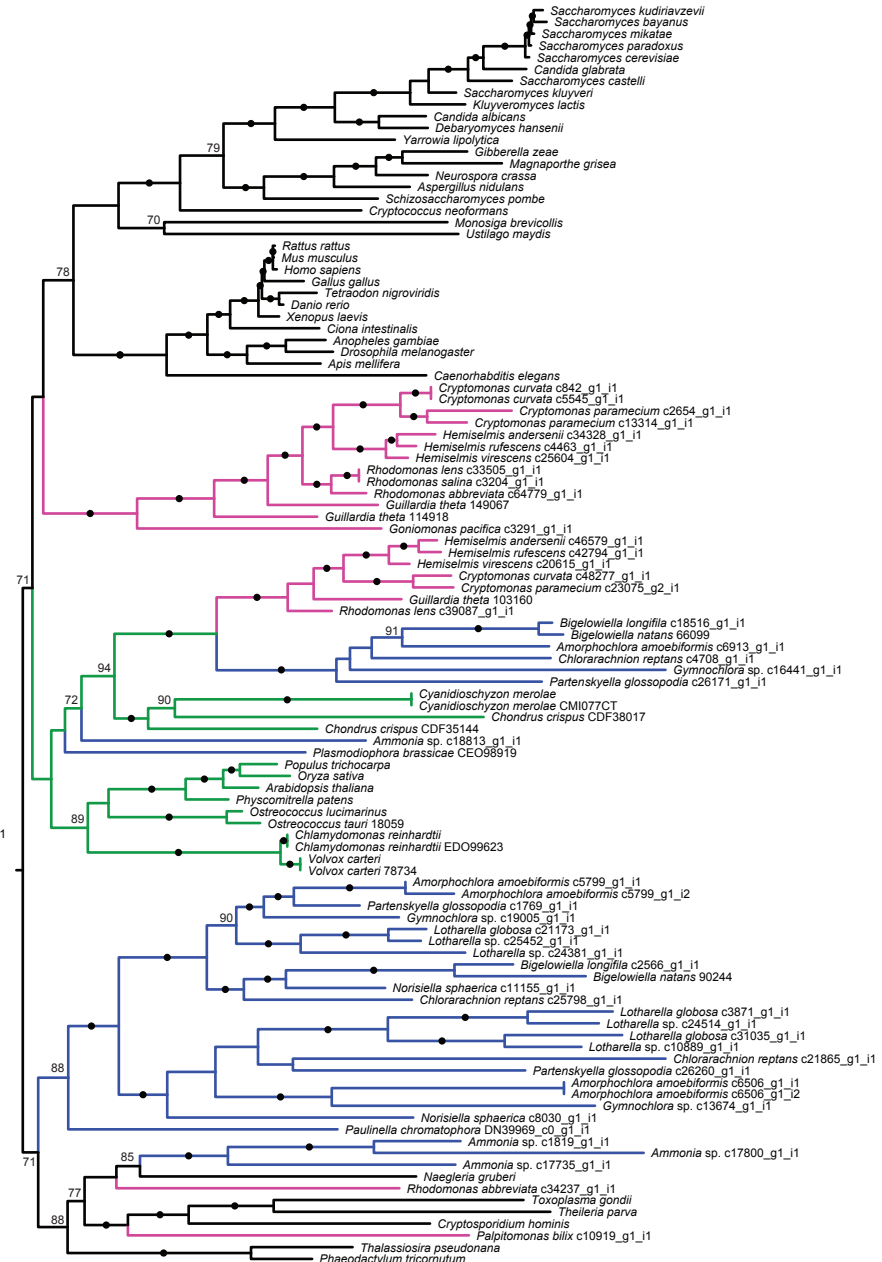

Supplement: Supplementary Data [file evz029_supp.zip › FigureS2.pdf]
